# The Mating System of the Wild-to-Domesticated Complex of *Gossypium hirsutum* L. Is Mixed

**DOI:** 10.3389/fpls.2018.00574

**Published:** 2018-05-09

**Authors:** Rebeca Velázquez-López, Ana Wegier, Valeria Alavez, Javier Pérez-López, Valeria Vázquez-Barrios, Denise Arroyo-Lambaer, Alejandro Ponce-Mendoza, William E. Kunin

**Affiliations:** ^1^Laboratorio de Genética de la Conservación, Jardín Botánico, Instituto de Biología, Universidad Nacional Autónoma de México, Mexico City, Mexico; ^2^Comisión Nacional para el Conocimiento y Uso de la Biodiversidad, Mexico City, Mexico; ^3^Department of Ecology and Evolution, Faculty of Biological Sciences, University of Leeds, Leeds, United Kingdom

**Keywords:** cotton, crop wild relatives, mating system, reproductive success, xenogamy, autogamy, domestication process, introgression

## Abstract

The domestication syndrome of many plants includes changes in their mating systems. The evolution of the latter is shaped by ecological and genetic factors that are particular to an area. Thus, the reproductive biology of wild relatives must be studied in their natural distribution to understand the mating system of a crop species as a whole. *Gossypium hirsutum* (upland cotton) includes both domesticated varieties and wild populations of the same species. Most studies on mating systems describe cultivated cotton as self-pollinated, while studies on pollen dispersal report outcrossing; however, the mating system of upland cotton has not been described as mixed and little is known about its wild relatives. In this study we selected two wild metapopulations for comparison with domesticated plants and one metapopulation with evidence of recent gene flow between wild relatives and the crop to evaluate the mating system of cotton’s wild-to-domesticated complex. Using classic reproductive biology methods, our data demonstrate that upland cotton presents a mixed mating system throughout the complex. Given cotton’s capacity for outcrossing, differences caused by the domestication process in cultivated individuals can have consequences for its wild relatives. This characterization of the diversity of the wild relatives in their natural distribution, as well as their interactions with the crop, will be useful to design and implement adequate strategies for conservation and biosecurity.

## Introduction

Plant domestication is a complex and continuing process (Casas et al., 2007; Vaughan et al., 2007). For 10,000 years, humans have selected attributes of interest in a range of economically valuable plants through their management and utilization (Gepts, 2004); consequently, different techniques, trait preferences, environments, and selection intensities have shaped the degree of domestication of each species (Meyer and Purugganan, 2013). Today we can find: (1) crop populations that are highly domesticated and depend on human intervention for survival; (2) semi-domesticated populations with recognizable traits of the domestication syndrome, but able to survive in the wild if human intervention ceases; (3) incipiently domesticated populations whose selected traits have not yet diverged markedly from those found in wild populations; (4) incidentally co-evolved populations that adapt to human disturbed environments, but without direct human selection; (5) feral populations derived from 2, 3, or 4; and (6) wild relatives (Clement, 1999). Given these diverse scenarios, the biological diversity contained in wild-to-domesticated complexes should be considered in studies about crop ecology and evolution (Warwick and Stewart, 2005; Casas et al., 2007).

In plants, one of the key life history traits is the mating system (Vaughan et al., 2007). This feature helps determine the genetic composition of populations and, therefore, has a crucial role in the evolution of species (Charlesworth, 2006); additionally, it explains who is mating with whom, which is a fundamental issue for conservation biology (Barrett and Harder, 1996). The mating system often changes during domestication (Meyer et al., 2012), and wild relatives contain the plesiomorphic state of this trait (Ellstrand et al., 1999; Doebley et al., 2006; Andersson and de Vicente, 2010). A shift from the ancestral system toward a new one can be selected until fixation; for instance, there are some crops that are unable to reproduce without human intervention (Ellstrand et al., 1999), such as vegetatively propagated sycamore fig and other fruit trees (Zohary and Spiegel-Roy, 1975). Some crops have multiple mating systems, such as domesticated ‘Maradol’ (*Carica papaya*), which is hermaphroditic, while native varieties and wild papayas are dioecious (Carvalho and Renner, 2012). Importantly, the characterization of the mating system of many plant species has been biased toward the domesticated counterparts, because only a sub-sample of the wild-to-domesticated complex was used (e.g., *Carica papaya* (Damasceno et al., 2009), *Persea americana* (Ish-Am et al., 1999), *Piper nigrum* (Thangaselvabal et al., 2008). This bias may have profound consequences for the conservation of conspecific wild relatives, especially because conclusions drawn from studies with domesticated varieties are extrapolated to the whole species, failing to consider the genotypic and phenotypic diversity that wild relatives possess. The conservation of this diversity is fundamental, because it is a genetic reservoir that includes a wider range of adaptive traits that may be of additional agricultural relevance, such as resistance to pests and pathogens and tolerance to abiotic stresses (Warschefsky et al., 2014).

Upland cotton, *Gossypium hirsutum*, is an economically important plant species, particularly known for being the leading source of natural fiber. Worldwide, over 90% of cotton production comes from cultivars of *G. hirsutum* and in 2014 the species ranked eighth in the world’s harvested area, reaching almost 35 million hectares (Crop production, FAOSTAT, 2017). Given the economic importance of the species, its mating system has been the focus of several studies since 1903 (Simpson, 1954); however, the majority of them concentrated on domesticated cotton and have described it as predominantly autogamous and self-pollinated (see Supplementary Material 1). On the other hand, studies on pollen dispersal of *G. hirsutum*, from the beginning of its modern breeding as a crop to the present day, refer to cotton’s ability to produce offspring by crossing (1944–2016; see Supplementary Material 1 for a review); however, the mating system is not described as mixed (Loden and Richmond, 1951; Richmond, 1951; Simpson, 1954; Imam and Allard, 1965; Meredith and Bridge, 1973). A specific study on the mating system of wild populations in their natural distribution is lacking.

In Mesoamerica, *G. hirsutum* exists as a complex of wild to domesticated forms (Brubaker and Wendel, 1994); hence, it is an ideal region to characterize the mating system of this upland cotton complex, identify possible differences, and to integrate this information into regional management plans. In Mexico - its center of origin, diversity and domestication (Ulloa et al., 2005; Burgeff et al., 2014; Pérez- Mendoza et al., 2016) – the complex includes cultivated and highly improved varieties, genetically modified varieties, traditionally managed landraces, feral, and wild populations. All of them belong to the primary gene pool of the species (Andersson and de Vicente, 2010) and gene flow among them occurs, even over long distances (Wegier et al., 2011). Moreover, eight wild *G. hirsutum* metapopulations have been recognized, based on geographic, ecologic, and genetic differences (Wegier et al., 2011; Bauer-Panskus et al., 2013). Wegier et al. (2011) demonstrated that recent gene flow, followed by introgressive hybridization, occurs between a number of wild populations distributed in the north and south of Mexico, and commercial cotton cultivars in the northern states of the country. Our study provides the first data on the mating system of wild *G. hirsutum in situ* within the natural distribution of the species in Mexico. In order to assess this, we evaluated the capacity of domesticated cotton, wild cotton, and wild cotton with evidence of introgression, to produce offspring by either xenogamy (cross-pollination between different genets) or autogamy (self-fertilization).

## Materials and Methods

### Study System

Upland cotton, *G. hirsutum* L., is a species with wild, feral, and semi-domesticated populations (Brubaker and Wendel, 1994). All cultivated forms, including the highly improved varieties or genetically engineered varieties, cannot be considered as fully domesticated, because they are able to survive even if human intervention stops.

Wild *G. hirsutum* flowers all year round. Flowers are white, hermaphrodite, cup shaped, with a single central style surrounded at the bottom by stamens (Meade, 1918; Smith and Cothren, 1999). Some plants exhibit flowers with a colored disk inside of the base of the cup that ranges from deep red to light yellow (Tan et al., 2013). Flowers remain open between 8 and 11 h; at the start of the day, they are all white and when they close the sepals start turning pink at the base (Smith and Cothren, 1999). Anthesis takes place in the morning, as soon as the flower completely opens, and the stamens start to release pollen soon afterward (Smith and Cothren, 1999). Flowers produce both pollen and nectar as a reward for visitors (Wäckers and Bonifay, 2004).

### Study Sites

**FIGURE 1:**
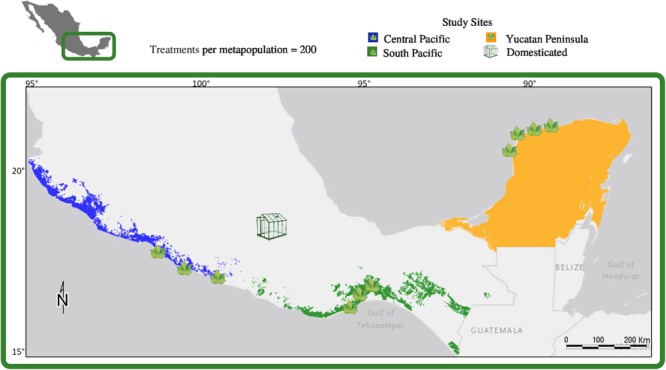
Approximate distributions of the Central Pacific (blue), Southern Pacific (green) and Yucatan Peninsula (orange) upland cotton metapopulations studied in Mexico, with locations of field study sites (leaves) and the greenhouse in Mexico City where cultivated upland cotton was grown out for this study. The Central Pacific and Yucatan Peninsula metapopulations are wild and the Southern Pacific metapopulation contains populations with evidence of recent introgression with cultivated upland cotton.

Sampling was performed in coastal dunes and dry forests of Mexico, in three of the eight wild cotton metapopulations defined genetically, geographically, and ecologically by Wegier et al. (2011), namely: Central Pacific Metapopulation (CPM), Yucatan Peninsula Metapopulation (YPM), and South Pacific Metapopulation (SPM). SPM is of particular interest due to evidence of recent introgressive hybridization with domesticated plants (Wegier et al., 2011). Given the distinctive extinction-colonization dynamic observed in cotton metapopulations (Wegier, 2013), the full extent of CPM, YPM, and SPM was surveyed to find *G. hirsutum* patches with enough flowers (**Figure 1**). Hand-pollination treatments (Tate and Simpson, 2004; Machado and Sazima, 2008; Hernández-Montero and Sosa, 2016) were carried out during the dry season, between November 2012 and May 2013: CPM in November 2012, YPM in December 2012, and SPM in February 2013. Sites were revisited for fruit collection after 86 days, on average. In addition, domesticated cotton plants, bought in local markets, were kept under greenhouse conditions in Mexico City to maintain a suitable temperature (**Figure 1**).

### Mating System

In order to execute the hand-pollination treatments to test for different mating systems, a search was conducted for flower buds before anthesis (Tate and Simpson, 2004; Machado and Sazima, 2008; Hernández-Montero and Sosa, 2016). In each metapopulation, 40 replicates of the five pollination treatments were set up (**Table 1**), anticipating the risk of collecting too few fruits afterward: assisted self-pollination, automatic self-pollination, assisted cross-pollination (Kearns and Inouye, 1993), emasculated control (to avoid automatic self-pollination), and control (open-pollination). Multiple treatments were placed on the same plant where possible to control for individual variation; however, due to the variability of the number of flowers, not all of the plants held the same type or number of treatments. Moreover, when flowers were scarce, treatments were placed daily within each study site, in up to four patches per metapopulation, until the 40 replicates per treatment were completed. Special care was taken to avoid changing or altering the environment (i.e., without introducing new genotypes or changing plant abundances or distributions). The same experimental design was applied for domesticated plants in a greenhouse that allowed the entry of local insects. Some treatments required mesh bags to exclude any pollinator access that could alter the results (**Table 1**). The treatments that did not include bagging before anthesis were bagged after flower closure to help control for mechanical damage from bagging.

**Table 1:** Characteristics of each of the pollination treatments applied to *G. hirsutum* flowers, following Dafni (1992), in three field wild metapopulations and cultivated cotton in the greenhouse.

Hand Treatment	Pollination type	Emasculation	Bag	Pollen source
Automatic self-pollination	Autogamy	No	Yes	Bagged before anthesis and no hand pollination provided.
Assisted self-pollination	Autogamy	No	Yes	Hand pollinated from the anthers to the stigma of the same flower; bagged before anthesis, and after hand self-pollination.
Assisted cross-pollination	Xenogamy	Yes	Yes	Pollen from a different plant was transferred to the stigma of the emasculated focal flower before bag placement.
Emasculated Control	Allogamy (xenogamy and geitonogamy)	Yes	No	Flower emasculated and no hand pollination done; bagged only after closing.
Open-pollination Control	Autogamy and allogamy	No	No	No hand pollination or emasculation of the flower; bagged only after closing.

### Reproductive Success

Fruit-set was calculated as the percentage of recorded fruits produced by each treatment in each metapopulation (Dafni, 1992). In each study site, 20 flowers not involved in the pollination-treatments were collected and brought back to the laboratory in separate sealed containers with 70% alcohol. Each flower was dissected to count the number of ovules present. An average number of ovules was calculated for each wild metapopulation and for domesticated plants. Afterward, seed-set (Schoper et al., 1987; Burd, 1994) was calculated as the percentage of seeds obtained from each fruit for each pollination treatment in relation to the average number of ovules of the study population to which these fruits belonged. Additionally, all seeds were weighed individually to estimate the seed weight per treatment in each study site. Later, all the seeds were germinated individually. Each seed was washed with 2% Captan (PESTANAL^®^, Merck) solution and covered with a damp cotton swab; tissue culture lids were used. Seeds were checked daily until all reached emergence of the radicle. While some studies on seed germination consider only a set of seeds (Schemske, 1983; Gil and López, 2015; Raphael et al., 2017; Farooq et al., 2018), we took into account all of the collected seeds for the analysis.

### Outcrossing Rate

The outcrossing rate (*Te*) was calculated for each study site following Barrett et al. (1996):

(1) $$\begin{array}{c} Te\;=\;1-S \end{array}$$

where *S* is the selfing rate, estimated with the fruit-set results from our selfing (*Ws*) and outcrossing (*Wx*) treatments, i.e., automatic self-pollination and emasculated control, respectively. For CPM, *Wx* was obtained with the fruit-set from the assisted cross-pollination treatment, because none of the emasculated control results were found when revisiting the metapopulation for fruit collection.

(2) $$\begin{array}{c} S\;=\;(\frac{w_{s}}{w_{s}+w_{x}}) \end{array}$$

### Statistical Analyses

To test if there were significant differences in seed-set and seed weight among treatments, a Generalized Linear Mixed Model GLMM (Zuur et al., 2009) was used considering the plant as a random factor, because the pollination treatments were not equally represented in each plant (as explained in section 2.3). For GLMM analyses, a Quasi-Poisson distribution was considered for seed-set and a Gaussian distribution for seed weight (Cayuela, 2009). Afterward, a Tukey *post hoc* test was performed to evaluate the significance of the results. To compare germination frequencies and percentage of fruit-set, a chi square test was used with the *post hoc* standardized residue test for each one. Outliers were identified using the method described by Viechtbauer and Cheung (2010); to summarize, a multivariate detection method (Cook distance) was used to calculate the distance among all data points, and those that were not included in the general model were identified as “influential data points” or outlier values. Germination was calculated as the number of germinated seeds in relation to the total number of seeds (Gómez, 2004; Gil and López, 2015). All tests were carried out with the *lme4, multcomp, stats*, and *ggplot2* packages of R version 3.4.3 (R Core Team, 2017). The scripts utilized for the analyses are available online at https://github.com/conservationgenetics/BiologiaReproductiva.git.

## Results

### Fruit-Set, Seed-Set, and Seed Weight

All treatments produced fruits regardless of the metapopulation (**Table 2**). The CPM open-pollinated control showed the highest value of all treatments among all groups; YPM showed the highest fruit-set produced by outcrossing, and the lowest by automatic self-pollination and control treatment. On the other hand, the highest fruit-set was observed for all the treatments in SPM, with exception of the open-pollination control.

**Table 2:** Fruit-set percentage in five pollination treatments applied to *G. hirsutum* and Chi-square test per treatment.

Sites	Open-pollination: control	Emasculated control	Assisted self-pollination	Automatic self-pollination	Cross-pollination
YPM	20.93^∗^	20.00	32.69^∗^	13.73^∗^	51.85^∗^
CPM	60.00^∗^	ND	20.69^∗^	25.93	24.14
SPM	48.84	78.26	73.33^∗^	69.23^∗^	54.05
Domesticated	43.48	10.00	60.00^∗^	34.09	19.05
Chi-square test	χ^2^ = 18.70, *df* = 3, *p* = 3.1 × 10^-4^	NA	χ^2^ = 37.68, *df* = 3, *p* = 3.3 × 10^-8^	χ^2^ = 47.69, *df* = 3, *p* = 2.46 × 10^-10^	χ^2^ = 26.78, *df* = 3, *p* = 6.5 × 10^-6^

*^∗^Categories significantly different using adjusted standardized residuals greater than 2.0 and less than -2.0 (χ^*2*^ test). ND: Fruit-set could not be determined because none of the treated flowers were found when revisiting the metapopulation for fruit collection. NA: the chi-square test could not be determined because one of the treatments was not collected.*

**FIGURE 2:**
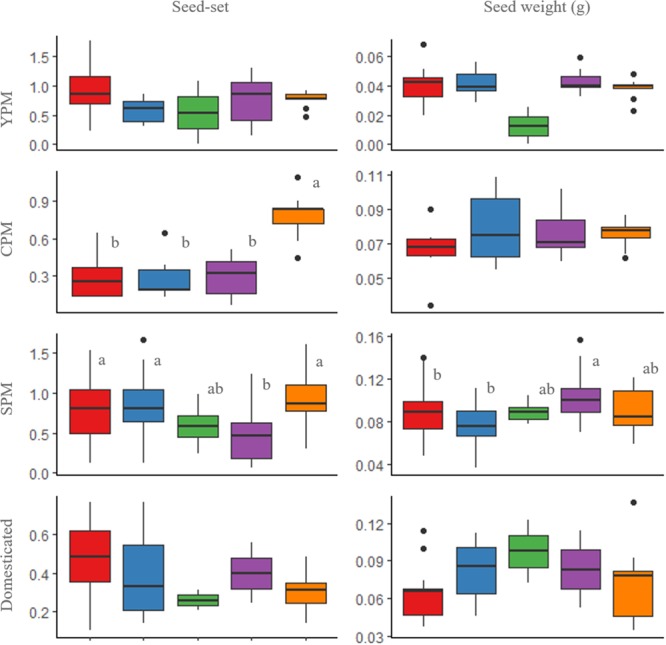
Box and whisker plots of seed-set and seed weight of the pollination treatments in wild upland cotton metapopulations (YPM and CPM), a metapopulation with recent gene flow (SPM), and domesticated plants. Treatments: assisted self-pollination (red), automatic self-pollination (blue), emasculated control (green), cross-pollination (purple), and open-pollination control (orange). The horizontal line within each box indicates the median. The bottom and top borders of the box are the first and third quartiles, respectively. The whiskers (vertical lines above and below the box) give the 99% range of the data; values outside this range are represented with a dot. Different letters indicate statistically significant differences identified by the Tukey test at *p* < 0.05.

**FIGURE 3:**
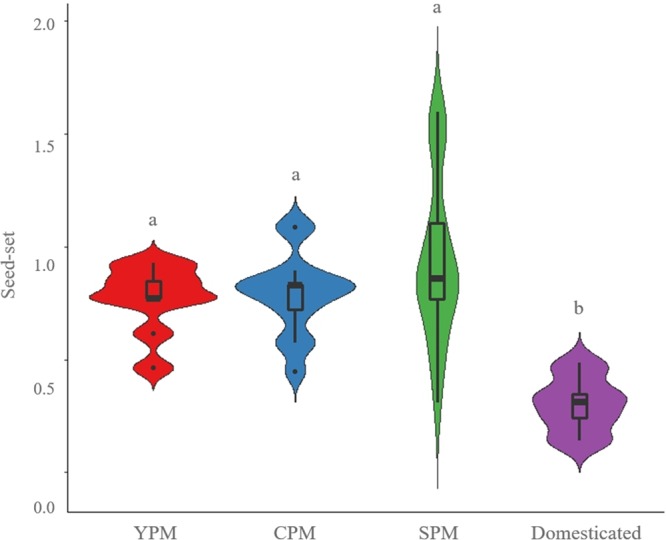
Violin plots showing seed-set densities of the control treatment (open pollination) in wild upland cotton metapopulations (YPM and CPM), a metapopulation with recent gene flow (SPM), and domesticated plants. The horizontal line within each box indicates the median. The bottom and top borders of the box are the first and third quartiles, respectively. The whiskers (vertical lines above and below the box) give the 99% range of the data; values outside this range are represented with a dot. Different letters indicate statistically significant differences identified by the Tukey test a *p* < 0.05.

Seeds were produced both by outcrossing and selfing treatments in all metapopulations (**Figure 2**). The average number of seeds per fruit was 15.9 in SPM, 15.4 in domesticated, 12.3 in CPM, and 10.0 in YPM, while the average number of ovules was 16.3 in SPM, 28.6 in domesticated, 15.6 in CPM, and 13.4 in YPM. Regarding seed-set, the control treatment of domesticated cotton was lower than that of wild and introgressed plants [*P*(χ^2^) = 4.13 × 10^-4^, *df* = 3] (**Figure 3**). When evaluating the results of each metapopulation individually, CPM presented seed-set differences between the control and the rest of the treatments (*P*(χ^2^) = 0.1 × 10^-4^, *df* = 3), in SPM the differences were found between cross-pollination and all treatments, except emasculated control [*P*(χ^2^) = 0.001, *df* = 4], while in YPM and the domesticated there were no significant differences among treatments (**Figure 2**). On the other hand, seed weight only presented differences in SPM, between cross-pollination and both assisted and automatic self-pollination [*P*(χ^2^) = 0.026, *df* = 4] (**Figure 2**). In YPM, CPM, and the domesticated cotton, no significant differences among treatments were observed (**Figure 2**).

### Germination

Less than 20% of the seeds from wild metapopulations CPM and YPM germinated. Regarding the domesticated group, 40–63% of the seeds germinated, except for the seeds produced by cross-pollination that only reached 28%. The seeds from the five treatments assessed at SPM showed germination percentages above 83%, with cross-pollination reaching the highest value of 96% (**Table 3**).

**Table 3:** Germination of seeds obtained from each upland cotton metapopulation and treatment.

Metapopulation	Traits	Germinated	Not germinated	Percentage of germination per treatment	Percentage of germination per metapopulation
**CPM+**	Assisted self-pollination	2	26	7.14	
χ^2^= 2.07, *df* = 3, *P* = 0.55	Automatic self-pollination	4	28	12.50	11.95
	Cross pollination	2	30	6.25	
	Open-pollination: control	25	159	13.59	
**YPM**	Assisted self-pollination	35	168	17.24	
χ^2^= 6.68, *df* = 4, *p* = 0.15	Automatic self-pollination	7	45	13.46	13.45
	Cross pollination	13	126	9.35	
	Open-pollination: Control	12	78	13.33	
	Emasculated control	0	14	0.00	
**SPM**	Assisted self-pollination	393	45	89.73	
χ^2^ = 19.29, *df* = 4, *p* = 0.0006	Automatic self-pollination	298	60^∗^	83.24	94.12
	Cross pollination	149	6^∗^	96.13	
	Open-pollination: Control	292	44	86.90	
	Emasculated control	150	25	85.71	
**Domesticated**	Assisted self-pollination	101	114	46.98	
χ^2^= 23.68, *df* = 4, *p* = 9.24 × 10^-5^	Automatic self-pollination	145	84^∗^	63.32	53.57
	Cross pollination	10^∗^	26^∗^	27.78	
	Open-pollination: control	53	40	56.99	
	Emasculated control	6	9	40.00	

*+All the repetitions of the emasculated control were placed, but none of them were found when revisiting the metapopulation for fruit collection. ^∗^Categories significantly different using adjusted standardized residuals greater than 2.0 and less than -2.0 (χ^*2*^ test).*

**FIGURE 4:**
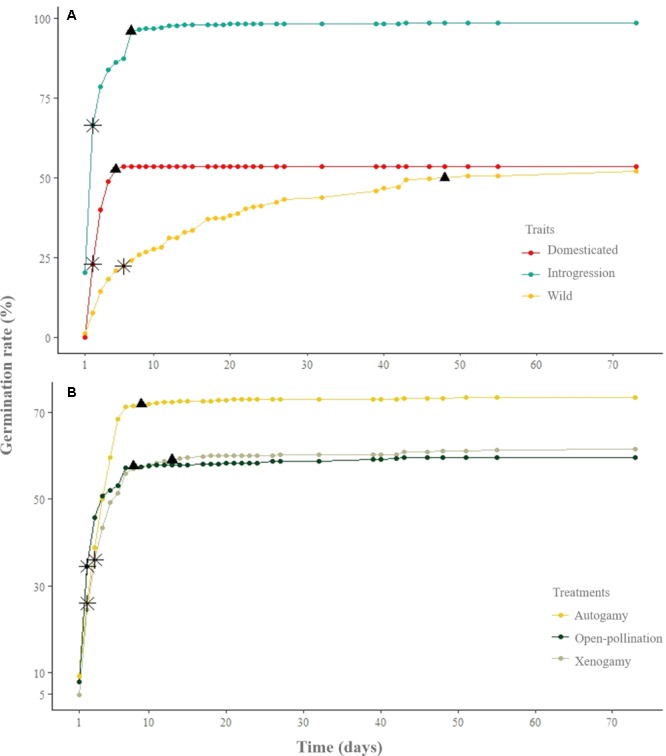
**(A)** Germination rate of seeds from domesticated plants, with introgressive hybridization plants (SPM), and wild plants (CPM and YPM). **(B)** Germination rate of pollination treatments from all study groups. The asterisk and the triangle represent 50 and 95 percent of the total of germinated seeds, respectively.

With regard to the germination rate of the seeds that germinated (**Figure 4**), the slope of the curve suggests that wild upland cotton presents some kind of inhibition to the completion of germination, whereas domesticated populations do not display this behavior. As shown in **Figure 4A**, domesticated seeds germinated faster, within the first 6 days, whereas seeds presenting evidence of introgressive hybridization (SPM) reached 95% of germination within the first 7 days and continued germinating for 48 days. Unlike domesticated and SPM seeds, the seeds of wild plants germinated over the course of 73 days (**Figure 4A**). Concerning the pollination treatments from all study sites, 50% of the seeds of all treatments germinated within the first 5 days; however, after the 5th day the difference in germination rate is evident between autogamy and the rest of the treatments (**Figure 4B**).

### Outcrossing Rate

All study groups presented outcrossing rates different from 0 and 1 (i.e., 1 > *Te* > 0), which is indicative of a mixed mating system. Wild metapopulations (YPM and CPM) recorded a higher outcrossing rate (0.72 and 0.71, respectively) than SPM (0.40) and domesticated (0.65) (Supplementary Material 1).

## Discussion

### Mating System of Upland Cotton’s Wild-Domesticated Complex

Richards (1997) defined autogamy as within-flower or self-pollination, and allogamy as the pollination between pollen and ovules of different flowers; moreover, he further divided allogamy into geitonogamy (i.e., pollination between different flowers on the same genet) and xenogamy (i.e., pollination between pollen and ovules of different genets). Our results show that wild and domesticated cotton produce offspring in all pollination treatments (**Figures 2, 4B** and **Tables 2, 3**); thus, the analyzed plants have the capacity to produce progeny by both autogamy and xenogamy. To discard geitonogamy, it is necessary to perform a molecular genetic analysis of paternity. However, since autogamy is common in our system, there is no need to discard this type of allogamy. Furthermore, previous studies (see Supplementary Material 1), together with our own, indicate that the *G. hirsutum* wild-domesticated complex has a mixed mating system. This result is particularly relevant in upland cotton’s center of origin, because of its significance on strategies for long-term conservation of genetic diversity in the event of gene flow between wild and domesticated relatives (Ellstrand, 1992).

Barrett and Eckert (1990) and Barrett et al. (1996) described the outcrossing rate (*Te*), which indicates that when the value is 0.5 the mating system is equally balanced between self and cross-pollination. Any value different from 0 (completely self-pollinated) or 1 (completely cross-pollinated) implies a mixed mating system; when *Te* > 0.5, the system is predominantly allogamous-xenogamous, whereas when *Te* < 0.5, the system is predominantly autogamous. Our observed rates vary from *Te* > 0.5, e.g., 0.71 (CPM), 0.72 (YPM) and 0.65 (domesticated), to *Te* < 0.5, e.g., 0.40 (SPM). Domesticated plants, and wild CPM and YPM, have a greater contribution of seeds from cross-pollination in the next generation, although the contribution of self-pollination is high and important, and it contributed to maintenance of genetic structure. The high contribution of self-pollinated seeds in SPM is striking, far from being similar or intermediate between wild and domesticated; local factors may be affecting the result and should be addressed in a future study.

To further explore the mixed mating system of the species, we compared the germination rate of seeds produced by different pollination treatments. We found that within the first 5 days the seeds for all treatments reach 50% of germination. After the 10th day, a notorious difference on germination rate (<15%) among autogamy and the other treatments is observed. Such discrepancy is due to the difference in number of seeds produced in each treatment (**Figure 4B**). As suggested theoretically, when germination does not differ among treatments, self-pollination is not the cause of inbreeding depression (Charlesworth and Willis, 2009). The mating system described in our study coincides with Baker’s law of reproductive assurance (Pannell and Spencer, 1998), where species that migrate long distances colonize or recolonize patches initially by self-fertilization; then, because of its perennial nature, generations overlap in the same area and plants are pollinated by close relatives or by themselves in the absence of pollinators (Kalisz et al., 2004). The information described here, agrees with the ecological and genetic evidence that describes the metapopulation dynamics of *G. hirsutum*, along with the ability to migrate long distances, historically and currently (Wegier et al., 2011).

In addition, Wegier et al. (2011) reported high values of gene flow among metapopulations in the same study area, which could homogenize genetic variation, but their data exhibit population structure (*k* = 8) and high *F*_ST_. Self-pollination and cross-pollination seem to maintain the genetic diversity of the species in the wild, although crossings with domesticated members of the complex (Wendel et al., 1992; Wegier et al., 2011) or even domesticated plants of *Gossypium barbadense* (Brubaker et al., 1993; Brubaker and Wendel, 1994; Ellstrand et al., 1999; Ellstrand, 2014) might be contributing to these results. In addition, gene flow with feral cotton can also take place (Rache Cardenal et al., 2013; de Menezes et al., 2015).

Finally, one of the fitness components measured in plants is seed weight (Primack and Kang, 1989), due to the fact that larger seeds perform better because of the higher amount of resources they possess (Armstrong and Westoby, 1993; Westoby et al., 1996). In our research, seed weight showed no significant difference between treatments within metapopulations (**Figure 2**).

### Differences of Reproductive Traits Within *G. hirsutum*’s Wild to Domesticated Complex

Our analyses show differences in characters linked to some of the reproductive structures of upland cotton, which can be associated with the domestication syndrome and will be discussed below.

#### Ovule Number

There are significant differences in ovule number [*P*(χ^2^) = 0.001, *df* = 2], which was initially estimated to obtain the seed-set in each population (Supplementary Material 2). Wild plants produce on average 14.5 ovules per flower, while cultivated plants produce twice as many. Several authors have described a change in ovule number as a consequence of evolutionary processes. For instance, Pasquet (1998) found that ovule number supports the physiological division of cultivated cowpeas [*Vigna unguiculata* (L.) Walp.] into two different groups: cultivars able to flower early under inductive conditions, with ovule number lower than 17 (*Biflora* and *Melanophthalmus*) and cultivars not able to do so, with ovule number higher than 17 (*Unguiculata* and *Sesquipedalis*). Moreover, Andargie et al. (2014) reported a pair of quantitative trait loci (QTLs; *qon1* and *qon3*) that regulate ovule number in cowpea; the alleles from the wild parent increase this trait as opposed to the cultivated, which reveals a feature of cowpea’s domestication syndrome. In the case of climbing common bean (*Phaseolus vulgaris* L.), among the changes that occurred during the domestication process is the modification on the number of ovules, which changed from 5–8 to 2–9 ovules (Gepts and Debouck, 1991).

#### Seed-Set

As shown in **Figure 3**, there are significant differences in the seed-set of wild and domesticated populations. From a much larger number of ovules, domesticated plants (open pollination controls) produce, proportionally, a lower quantity of seeds, which implies that they are not efficiently using the resources invested on ovule production (Cilas et al., 2010). Variation in seed number per boll is produced by the interplay of the plant genetics and the environment, which in turn generates either the lack of seed fertilization or completion of embryo growth post-fertilization (Davidonis et al., 1996); therefore, our results are influenced by the experimental design and, in the future, a common garden experiment will provide insight into the effect of the environment. In comparison, wild plants are more efficient, producing seeds from nearly all of their ovules, although the net number of seeds is smaller than that produced by domesticated fruits. Many features associated with domestication are not advantageous in terms of reproduction and survival of following generations lacking human intervention (Gepts, 2004), because the selective pressures by which they have evolved are determined by humans (see categories 1–4 of the classification proposed by Clement, 1999). As a result, gene flow between wild relatives and cultivated plants could have negative consequences (Andersson and de Vicente, 2010), however, it could also give rise to *in situ* reservoirs of domesticated genes for the future (Ellstrand, 2018). Each domesticated cotton plant develops 50% more descendant plants than the wild plants do within their natural distribution, so the ecological-evolutionary consequences of this result will depend on the evolutionary process and the agro-ecological or ecosystem context in which plants are developed.

#### Germination

One of the traits selected for during domestication is rapid germination (Frary and Doganlar, 2003), as this helps crops to start to grow at the same time and contributes to synchronous fruiting. Over time, this trait contributes to harvesting efforts and, therefore, unconsciously selects for loss of dormancy. In natural habitats, conditions are less predictable, and dormancy will contribute to different seeds germinating in different environmental conditions (Long et al., 2015). Our results on seeds that reached germination agree with what has been described for other domesticated plants that have undergone similar evolutionary processes (Fuller and Allaby, 2009; Abbo et al., 2014; Hernández et al., 2017): domesticated seeds germinate faster and practically simultaneously, whereas their wild relatives display dormancy (**Figure 4A**).

#### Distinctive Traits of SPM

With respect to SPM (selected for study because of evidence of recent introgression with domesticated plants; Wegier et al., 2011), the reproductive system is mixed, as it is in wild populations without introgression and in domesticated populations. However, some of the traits that determine reproductive success are unique to this population: the variability in seed-set values is markedly different (**Figures 2, 3**); its fruits produce more seeds than the other populations (similar to domesticated fruits, but from half the number of ovules, which makes them very efficient) (Supplementary Material 2); and these seeds have a higher percentage of germination than the other populations (**Table 3**). These characteristics can have demographic consequences in the short term, unless there are other factors that regulate this growth. On the other hand, contrary to what was expected for SPM, their resemblance to domesticated seed germination is higher than with the wild ones. The behavior is also dissimilar for introgressed seeds, which took longer to complete germination than domesticated and wild seeds (**Figure 4A**). This last phase displays a very slow response in SPM, probably associated with the loss of physiological responses, resembling domesticated plants. It is important that these analyses are repeated in subsequent years, to confirm if there is an eco-genetic trend (Price and Waser, 1979; Edmands, 2007; Ellstrand and Rieseberg, 2016), or if it was the result of local conditions.

### Conservation and Biosecurity Implications

Many nations want to defend the rights of the next generations to enjoy and decide about biodiversity and its services, aware that decisions made today will have an impact on the natural resources available in the future (Bennett et al., 2015; Steffen et al., 2015; Morales et al., 2017). Upland cotton is a remarkably important plant for humanity, not only due to the versatile uses of its fiber, but for many other applications (Wegier et al., 2016). It follows that cotton’s wild-to-domesticated complex and its environment should be a conservation priority. Mesoamerican dry forests and coastal dunes contain the ecosystems and evolutionary processes that originated, mold, and maintain wild cotton diversity and its interactions. These evolutionary services (Faith et al., 2010; Bailey, 2011; Rudman et al., 2017) are essential for species conservation, because preserving this genetic diversity allows the capacity to adapt to environmental changes (Ellstrand, 1992; Hartl, 2000). However, the factors that mold each part of the wild-to-domesticated complex are different; for example, the conservation of native traditional varieties depends to a great extent on the communities that cultivate them, their management techniques, and interests (Zhang et al., 2007). Hence, the parts of the complex that could be used for crop improvement will depend on the objectives of the new processes of domestication and breeding (Ellstrand, 2018; Mastretta-Yanes et al., 2018).

Gene flow between crops and wild relatives should be examined on a case-by-case basis (Stewart et al., 2003), especially when genetically modified organisms (GMO) are involved, because the consequences depend on the nature of the transferred genes and their regulatory mechanisms (Ellstrand, 2003). For instance, a recent study has demonstrated that genetic modifications can affect fitness traits in the long-term (Hernández-Terán et al., 2017). An important issue to keep in mind is that for gene flow to occur, a crop must be within pollination distance of a compatible population (Ellstrand and Hoffman, 1990), but in the case of domesticated plants the distances can be shortened by human activities (Dyer et al., 2009; Wegier et al., 2011). Several studies have documented hybridization events between crops and their wild relatives; for instance, in the United Kingdom, one-third of the 36 species analyzed by Raybould and Gray (1993) hybridize with at least one element of the local flora; in the Netherlands, a quarter of 42 species does (de Vries et al., 1992); and all but one of the 13 crops reviewed by Ellstrand et al. (1999) hybridize naturally with their wild relatives in some part of their agricultural distribution (including *G. hirsutum* and other species of subgenus *Karpas*). These hybridization events could lead to a decline in wild genetic diversity, as opposed to native semi-domesticated varieties in traditional Mesoamerican systems where there is evidence that domesticated genomes have formed not only by selection under domestication, but also by gene flow with other closely related populations and species (Rendón-Anaya et al., 2017). For this reason, the wild-to-domesticated dynamics in terms of genetic diversity, reproductive biology, and gene flow should be well understood in the natural distribution of the species of interest, because extrapolating conclusions based on external or incomplete information about species complexes is inconsistent with the objectives of conservation and biosafety (Beebe et al., 1997; Acevedo et al., 2016).

In this study we found that the reproductive capacity of introgressed cotton is greater than that of wild and domesticated plants. This reveals a scenario that de Wet (1968), de Wet and Harlan (1975) and Keeler et al. (1996) had already described, where wild relatives of some introgressed crops can become weeds that are difficult to control. The wide genetic diversity of *G. hirsutum*, along with factors modified by traditional genetic improvement and modern genetic engineering, will be problematic for agroecosystems (Altieri, 2000) and ecosystem conservation if they increase cotton’s weediness or invasiveness (Schafer et al., 2011). Cotton has already been reported to persist in a few tropical regions, such as the north of Australia, Vietnam, México, the continental United States and Hawaii (Hawkins et al., 2005; Andersson and de Vicente, 2010; USDA, 2018), so it will be necessary to monitor these changes in wild populations given the species great capacity for long distance migration by natural and anthropogenic means.

Finally, local conditions can influence the results of reproductive biology studies (Ellstrand and Foster, 1983; Hucl, 1996; Murray et al., 2002); hence, it was essential to assess the mating system of *G. hirsutum* within its natural distribution. Some of the factors that have an effect on the results can be associated with the environment (pollen viability, nectar production, and pollinator activity due to environmental conditions; Ahrent and Caviness, 1994; Ibarra-Pérez et al., 1997; Chaves-Barrantes et al., 2014), ecological interactions (foraging rate, floral consistency, efficiency of pollen deposition, interactions with arthropodofauna, and composition of pollinator species; Rudgers, 2004; Kessler et al., 2012; Johnson et al., 2015), as well as the landscape (species abundance and surrounding species distributions; Murray et al., 2002). In this study, the results of automatic self-pollination and the emasculated control provide evidence that autogamy and allogamy occur naturally in upland cotton’s natural distribution. The occurrence of the latter highlights the importance of native pollinators on the reproductive biology of *G. hirsutum* and, consequently, conservation strategies should take this key interaction into consideration.

## Conclusion

This study found that upland cotton’s wild-to-domesticated complex presents a mixed mating system. This information is new for wild, domesticated, and introgressed *G. hirsutum* in its natural distribution, but it is in agreement with previous studies in populations of domesticated cotton (Supplementary Material 3). Consequently, *G. hirsutum* should be considered as having a mixed reproductive strategy throughout its whole complex, rather than being primarily autogamous. Management strategies and policies meant to conserve the diversity of cotton’s wild-to-domesticated complex must take this into account.

Furthermore, physiological differences were found between cultivated cotton and its wild relatives, especially in traits such as the number of ovules per flower, number of viable seeds per fruit, and their germination behavior. Given the evidence of gene flow and introgression, these traits should be monitored systematically in wild populations and agroecosystems of interest for conservation, as well as the impact on ecological interactions, such as pollination. On the other hand, the diversity contained in the wild-to-domesticated complex must be included in long-term conservation strategies, so that future generations can have access to genetic resources with greater chances of surviving the changing environments.

## Author Contributions

AW, VA, and RV-L designed the research, participated in fieldwork, performed the analyses, and wrote the manuscript. AW coordinated the study. WK designed the fieldwork and revised the analyses. AP-M participated in fieldwork and performed the analyses. JP-L, VV-B, and DA-L conducted the analyses. All authors analyzed the results and wrote the manuscript.

## Conflict of Interest Statement

The authors declare that the research was conducted in the absence of any commercial or financial relationships that could be construed as a potential conflict of interest.
