# Supplementary material for: The Mating System of the Wild-to-Domesticated Complex of Gossypium hirsutum L. Is Mixed
Source: Front Plant Sci. 2018 May 9;9:574. doi: 10.3389/fpls.2018.00574 (PMC5954804; doi:10.3389/fpls.2018.00574)
Supplement: Supplementary file 1 [file Data_Sheet_1.docx]

Supplementary Material 1

The mating system of the wild-to-domesticated complex of *Gossypium hirsutum* L. is mixed

Rebeca Velázquez-López *^✚^, Ana Wegier *^✚^, Valeria Alavez, Javier Pérez-López, Valeria Vázquez-Barrios, Denise Arroyo-Lambaer, Alejandro Ponce-Mendoza, William E Kunin

^✚^ These authors contributed equally to this work.

*** Correspondence:** [rebecavelazquezl@gmail.com](mailto:rebecavelazquezl@gmail.com), [awegier@ib.unam.mx](mailto:awegier@ib.unam.mx)

For many years, the distance to which gene flow among domesticated cotton plots occurs has been a topic of interest for cotton specialists. Although the motivation to conduct experiments of this nature has been changing over time, there are numerous published studies from 1944 to 2016 (Table 1). Such studies quantify the outcrossing in remote plots over at least three progressive distances.

As part of the research presented here we have evaluated outcrossing rates in wild cotton localities, cultivated plants with and without introgression, and domesticated plants. We have taken into account both the biology of the species and our results, so we obtained and plotted the outcrossing rate of the aforementioned studies along with the data generated in our own study. Here we propose an Indirectly Estimated Outcrossing rate (IEO) that can be obtained from studies reporting outcrossing distance.

The efficiency and distribution of pollinators vary among different geographical areas and the interaction between wild and managed pollinators (e.g. Honey bees), so it is important to know if the results of the studies are comparable ([Garibaldi et al., 2013](#_ENREF_8)). Moreover, the use of insecticides can affect the presence and activity of pollinators, modifying the biological interpretation of the results ([Simpson, 1954](#_ENREF_16); [Kennedy et al., 2013](#_ENREF_9)). Another factor that could influence the results is the change in the process of genetic improvement of the crop, thus, the date on which the studies were published is also relevant ([Garibaldi et al., 2011](#_ENREF_7)). The IEO indicator can be correlated with these characteristics to recognize whether the effects of the insecticides are influenced by cross-over rate, time, space or presence of management.

**Methods**

We searched for papers, reviews, books, and book sections in databases including Google Scholar, Web of Science, and Scopus, with the following Boolean operators: (“crossing”) AND (“outcrossing”) AND (“gene flow”) AND (“*Gossypium hirsutum*”) OR (“cotton plant”). Subsequently, a nested search was carried out in the articles found to strengthen the scientific inquiry. Then, a database with outcrossing distances reported from the different studies was built; additional information, such as region where the study was developed, as well as records on the use of insecticide, were included.

The percentage of outcrossing from an area with marked plants with a recognizable feature to other areas located at progressive distances where this characteristic is not found was reported in all the selected studies. The percentage of outcrossing from the minimum distance (*a*) to the maximum distance (*n*) was plotted and a curve was adjusted, which may differ between models.

In order to be included in the analysis, the total outcrossing for each of the assessed treatments requires information about the outcrossing within the marked or the donor area. For this, the outcrossing percentage is estimated in *a´*, which is the distance reported from zero –the border between plots- until *a* within the donor plot. Then the known values of *a*, *b* and *c* (the first three distances, and the minimal data reported in all of the examined studies that enable comparisons) are added up. We have named the resulting rate the Indirectly Estimated Outcrossing rate (IEO), which is calculated as follows:

1. $\sum_{i=1}^{3} \left( f\left( i \right) \right)+a^{'}$

where *f(i)* is

1. $f\left( i \right)={ke}^{px}$

where *k* and *p* are constants coming from the adjustment of the negative exponential curve in each case; and *x* is the distance of the reported outcrossing for each study (*a*, *b* and *c*) (Figure 1).

In addition, to compare the IEO with our mating system analysis, the percentage of outcrossing was determined following Barrett et al. (1996):

(3) $Te=1-S$

where *S* is the selfing rate

(4) $S=(\frac{W_{s}}{W_{s}+W_{x}})$

where *Ws* and *Wx* are the offspring resulting from self-fertilization and outcrossing, taken from the fruit-set results of the emasculated control and cross-pollination treatments, respectively.


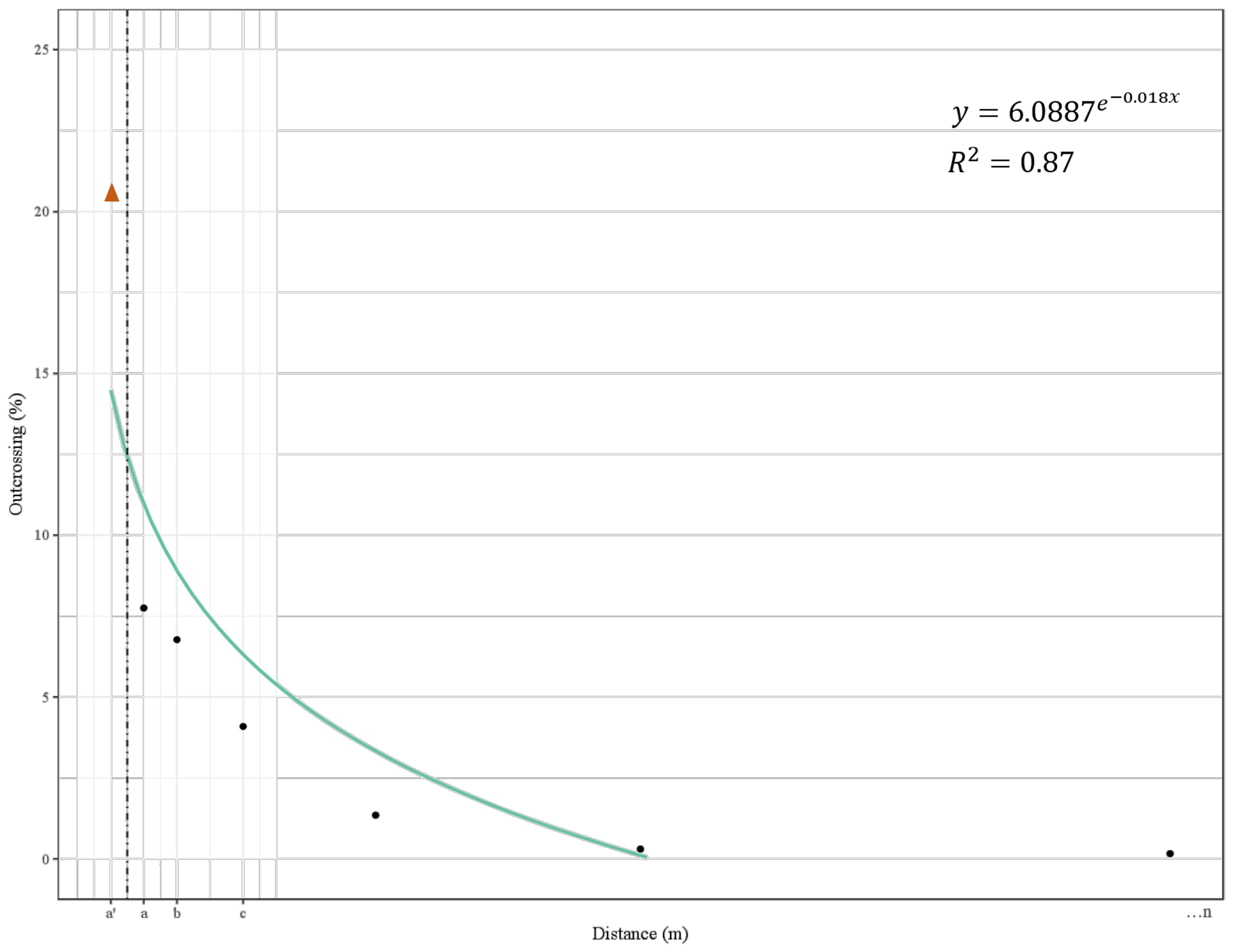


Figure 1. Outcrossing and the negative exponential trend line adjusted to the data from [Galal et al. (1972)](#_ENREF_6) (black dots). Outcrossing percentage between the donating plot and the first distance *a´* (orange triangle) (dotted line is the limit between plots).

Table 1. Results of the Indirectly Estimated Outcrossing rate (IEO) and methodological characteristics of the selected studies.

| **Site of study/Treatments** | **IEO / *Te*** | **Formula that describes the outcrossing between the donor plot and recipient plot** | **Insecticide** | **Region** | **Reference** |
| --- | --- | --- | --- | --- | --- |
| West Punjab, Pakistan | 31.5323 | $y=6.4045\times e^{-0.328\left( x \right)}$ | NO | Asian | [Khan and Afzal (1950)](#_ENREF_10) |
| West Punjab, Pakistan | 5.1823 | $y=0.4682 \times e^{-0.042 \left( x \right)}$ | NO | Asian | [Khan and Afzal (1950)](#_ENREF_10) |
| West Punjab, Pakistan | 10.1955 | $y=0.56261 \times e^{-0.05\left( x \right)}$ | NO | Asian | [Khan and Afzal (1950)](#_ENREF_10) |
| Iraq/hybridization | 5.8174 | $y=0.6995 \times e^{-0.084\left( x \right)}$ | NO | Asian | [Al Jibouri (1960)](#_ENREF_1) |
| Wad Medani, Sudan/ Outcrossing | 18.5097 | $y=6.3507 \times e^{-0.114\left( x \right)}$ | YES | African | [Ali et al. (2003)](#_ENREF_2) |
| Mississippi Delta, USA | 7.0854 | $y=5.0337 \times e^{-0.184\left( x \right)}$ | YES | American | [Berkey et al. (2002)](#_ENREF_3) |
| Alexandria, Egypt (Apiary East-West) | 11.2333 | $y=8.259 \times e^{-0.562\left( x \right)}$ | YES | African | [Elfawal et al. (1976)](#_ENREF_4) |
| Alexandria, Egypt (Apiary North-South) | 2.6680 | $y=-0.237\times e^{3.0333\left( x \right)}$ | YES | African | [Elfawal et al. (1976)](#_ENREF_4) |
| Alexandria, Egypt (Away from apiary East-West) | 2.0913 | $y=0.2194\times e^{-0.0568\left( x \right)}$ | YES | African | [Elfawal et al. (1976)](#_ENREF_4) |
| Alexandria, Egypt (Away from apiary North-South) | 24.1397 | $y=0.4482\times e^{-0.26\left( x \right)}$ | YES | African | [Elfawal et al. (1976)](#_ENREF_4) |
| Shalakan, Egypt/ cross-pollination | 28.3134 | $y=6.0887\times e^{-0.118\left( x \right)}$ | YES | African | [Galal et al. (1972)](#_ENREF_6) (Figure 1) |
| Kununurra, Australia | 14.4092 | $y=3.6887\times e^{-0.04\left( x \right)}$ | YES | Australian | [Llewellyn et al. (2007)](#_ENREF_12) |
| Kununurra, Australia | 67.6614 | $y=14.922\times e^{-0.056\left( x \right)}$ | YES | Australian | [Llewellyn et al. (2007)](#_ENREF_12) |
| Namoi Valley, Australia/ randomized plot of transgenic and non-transgenic cotton plants | 0.4354 | $y=0.2054\times e^{-0.314\left( x \right)}$ | YES | Australian | [Llewellyn and Fitt (1996)](#_ENREF_11) |
| Andalusia, Spain/ pollen mediated gene flow between GM and non-GM across different directions (2007) | 5.6496 | $y=1.1917\times e^{-0.131\left( x \right)}$ | YES | European | [Loureiro et al. (2016)](#_ENREF_13) |
| Andalusia, Spain/ pollen mediated gene flow between GM and non-GM across different directions (2009) | 0.4186 | $y=0.1396\times e^{-0.199\left( x \right)}$ | YES | European | [Loureiro et al. (2016)](#_ENREF_13) |
| Andalusia, Spain/ pollen mediated gene flow between GM and non-GM across different directions (2010) | 1.4258 | $y=0.2371\times e^{-0.038\left( x \right)}$ | YES | European | [Loureiro et al. (2016)](#_ENREF_13) |
| Tennessee, USA (barrier of three rows of corn) | 88.76025 | $y=21.208\times e^{-0.036\left( x \right)}$ | NO | American | [Pope et al. (1944)](#_ENREF_14) |
| Tennessee, USA (barrier of six rows of corn) | 71.5659 | $y=13.566\times e^{-0.0106\left( x \right)}$ | NO | American | [Pope et al. (1944)](#_ENREF_14) |
| Tennessee, USA (barrier of nine rows of corn) | 67.2158 | $y=9.5291\times e^{-0.0302\left( x \right)}$ | NO | American | [Pope et al. (1944)](#_ENREF_14) |
| Tennessee, USA (without barrier) | 136.4394 | $y=27.468\times e^{-0.005\left( x \right)}$ | NO | American | [Pope et al. (1944)](#_ENREF_14) |
| Kahramanmaras, Turkey | 64.4377 | $y=14.914\times e^{-0.586\left( x \right)}$ | NO | Asian | [Sen et al. (2004)](#_ENREF_15) |
| Tennessee, USA | 201.8321 | $y=15.362\times e^{-0.095\left( x \right)}$ | NO | American | [Simpson and Duncan (1956)](#_ENREF_17) |
| Tennessee, USA | 66.3835 | $y=15.362\times e^{-0.095\left( x \right)}$ | NO | American | [Simpson and Duncan (1956)](#_ENREF_17) |
| Tennessee, USA | 72.5442 | $y=16.683\times e^{-0.1\left( x \right)}$ | NO | American | [Simpson and Duncan (1956)](#_ENREF_17) |
| Tennessee, USA | 552.2646 | $y=110.13\times e^{-0.356\left( x \right)}$ | NO | American | [Stephens and Finkner (1953)](#_ENREF_19) |
| Tennessee, USA | 383.5035 | $y=120.44\times e^{-0.143\left( x \right)}$ | NO | American | [Stephens and Finkner (1953)](#_ENREF_19) |
| Mississippi, USA/Gene flow from a GM cotton plot | 43.1752 | $y=6.8752\times e^{-0.098\left( x \right)}$ | YES | American | [Umbeck et al. (1991)](#_ENREF_20) |
| Mississippi, USA/Gene flow from a GM cotton plot | 31.0105 | $y=2.3398\times e^{-0.068\left( x \right)}$ | YES | American | [Umbeck et al. (1991)](#_ENREF_20) |
| California, USA/Detection of gene flow from herbicide-resistant to commercial cotton plots. | 12.8944 | $y=4.0211\times e^{-0.094\left( x \right)}$ | YES | American | [Van Deynze et al. (2005)](#_ENREF_21) |
| Thessaloniki, Greece /Outcrossing | 15.5137 | $y=3.639\times e^{-0.9074\left( x \right)}$ | YES | European | [Xanthopoulos and Kechagia (2000)](#_ENREF_24) |
| Thessaloniki, Greece /Outcrossing | 23.4479 | $y=3.8759\times e^{-0.263\left( x \right)}$ | YES | European | [Xanthopoulos and Kechagia (2000)](#_ENREF_24) |
| Shuo Yan, China/Apis pollinators 1er year | 23.89508379 | $y=5.1362\times e^{-0.049\left( x \right)}$ | YES | Asian | [Yan et al. (2015)](#_ENREF_25) |
| Shuo Yan, China/Bombus pollinators 1er year | 98.9381 | $y=20.474\times e^{-0.105\left( x \right)}$ | YES | Asian | [Yan et al. (2015)](#_ENREF_25) |
| Shuo Yan, China/Pieris pollinators 1er year | 13.5648 | $y=1.8788\times e^{-0.004\left( x \right)}$ | YES | Asian | [Yan et al. (2015)](#_ENREF_25) |
| Shuo Yan, China/Apis pollinators 1er year | 97.9353 | $y=23.52\times e^{-0.138\left( x \right)}$ | YES | Asian | [Yan et al. (2015)](#_ENREF_25) |
| Shuo Yan, China/Bombus pollinators 1er year | 149.1556 | $y=31.656\times e^{-0.095\left( x \right)}$ | YES | Asian | [Yan et al. (2015)](#_ENREF_25) |
| Shuo Yan, China/Pieris pollinators 1er year | 41.2839 | $y=7.5691\times e^{-0.062\left( x \right)}$ | YES | Asian | [Yan et al. (2015)](#_ENREF_25) |
| Shuo Yan, China/Apis pollinators 2er year | 23.6815 | $y=4.0204\times e^{-0.035\left( x \right)}$ | YES | Asian | [Yan et al. (2015)](#_ENREF_25) |
| Shuo Yan, China/Bombus pollinators 2er year | 76.4797 | $y=14.14\times e^{-0.057\left( x \right)}$ | YES | Asian | [Yan et al. (2015)](#_ENREF_25) |
| Shuo Yan, China/Pieris pollinators 2er year | 10.02 | $y=1.8788\times e^{-0.037\left( x \right)}$ | YES | Asian | [Yan et al. (2015)](#_ENREF_25) |
| Shuo Yan, China/Apis pollinators 2er year | 80.1553 | $y=13.092\times e^{-0.037\left( x \right)}\uparrow$ | YES | Asian | [Yan et al. (2015)](#_ENREF_25) |
| Shuo Yan, China/Bombus pollinators 2er year | 108.7731 | $y=14.995\times e^{-0.036\left( x \right)}$ | YES | Asian | [Yan et al. (2015)](#_ENREF_25) |
| Shuo Yan, China/Pieris pollinators 2er year | 39.8667 | $y=6.3216\times e^{-0.038\left( x \right)}$ | YES | Asian | [Yan et al. (2015)](#_ENREF_25) |
| Huanghe Valley, China/BT insect-resistant transgenic cotton | 18.3303 | $y=4.0871\times e^{-0.214\left( x \right)}$ | YES | Asian | [Zhang et al. (2005)](#_ENREF_26) |
| Huanghe Valley, China/ TFDA with resistance to herbicide | 21.2353 | $y=3.8355\times e^{-0.099\left( x \right)}$ | YES | Asian | [Zhang et al. (2005)](#_ENREF_26) |
| México, YPM | 72.1642 | $Te=1-s$  $s=\frac{W_{s}}{W_{s}+W_{x}}$ | NO | American | This study |
| México, PCM | 71.3097 |  | NO | American | This study |
| México, Domesticated | 65.5765 |  | NO | American | This study |
| México, SPM | 40.8510 |  | NO | American | This study |

**Results**

Of the studies analyzed (n = 53), 59 % were below the IEO average (40.74), 81 % of these studies used some type of insecticide in their experiments (Figure 2A). Regarding studies that are above the average, insecticides were used in 33 % of them; however, they recorded higher IEO values than those articles that, besides using insecticides, did not consider pollinators (Figure 2B and 2C).

We analyzed the geographical patterns (by continents) and agricultural management (pest control), and -given the wide dispersion of the data within and between each one of them- we only found significant differences with the papers published before 1960 and afterwards.


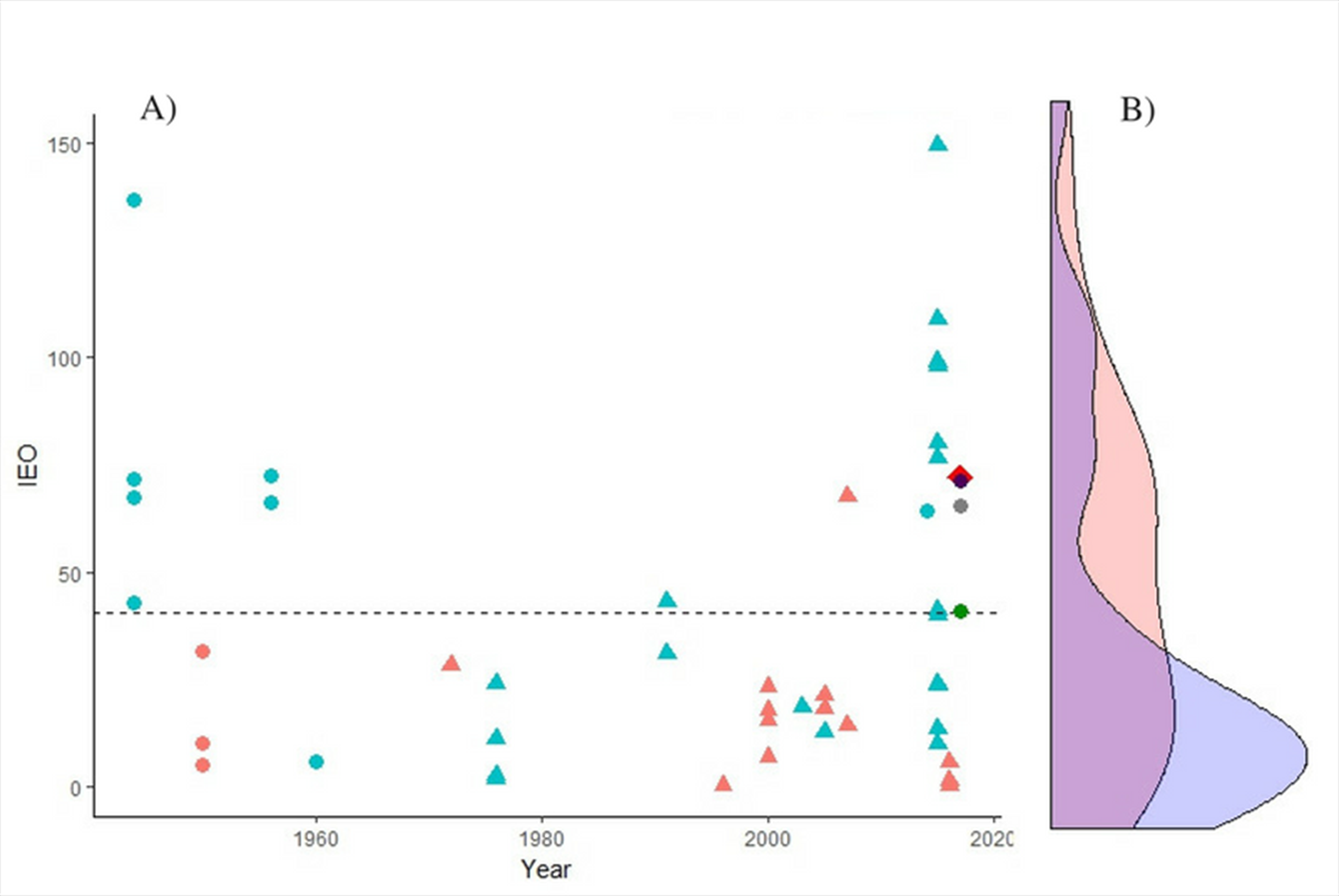


Figure 2. A) Indirectly Estimated Outcrossing rate (IEO) of the analyzed studies and the Outcrossing rate (*Te*) of our study. The shape denotes the use (triangle) or absence (circle) of insecticide. Studies taking into account pollinators in the methods, results or discussion are in blue, otherwise in red. Code color for the Outcrossing rate:  YPM (orange), CPM (navy) SPM (green) and domesticated plants (scarlet). The dotted line represents the average of IEO. B) Density analysis, presence of pollinators (pink) and use of insecticide data (violet).

**Discussion**

The studies measuring the pollen dispersal distance are plentiful for domesticated cotton (34 out of the 53 studies analyzed, Table 1). These studies may be used to compare local outcrossing in different geographic areas, time of year and repetitions among other variables by homogenizing the results with the IEO (Figure 1). It is worth noting that a large number of the studies were published before 1980 given the interest in improving the crop and after 2000 due to the interest for management of genetically modified cotton.

The outcomes of these analyses show variability that can be seen in the outcrossing rates of a mixed system, such as the one observed for cotton. The experimental design and the environmental conditions might strongly influence the results. Among the factors causing alterations are: shape and arrangement of plots, distance, measure of the distance, presence of barriers, pollinator exclusion, seasonality, agricultural management, and even the technical capabilities to detect outcrossing ([Simpson, 1954](#_ENREF_16)); nevertheless, our data was also influenced by methodological aspects as described in our manuscript.

There are two reasons to think that the data is still underestimated. Firstly, we only used the outcrossing data with marking of donor plots, which -within our analysis- precludes the outcrossing from unmarked plants located both nearby and further away. Secondly, only the first three quantified distances were used; however, the studies often suggest that the pollen contribution to outcrossing continues beyond such areas. Due to the fact that the data reported in the studies is highly variable, we were only able to assuredly obtain the first three data points. A possible solution to this is to estimate further distances similarly to *a´*, although we would then obtain the opposite effect: an overestimation of the data, as 59 % of the studies did not report outcrossing for at least one of the assessed distances.

As shown in Figure 2, our data is over the IEO average and is similar to those studies that did not use insecticide. More frequent interactions with pollinators within the center of origin would be expected, so that the outcrossing is present; nonetheless, when comparing with other studies, several sites have high rates of outcrossing and for most of the cases low IEO values are associated with insecticide management. On the other hand, the observation of pollinators on a plant is common among both low and high IEO values; therefore, it is our belief the type and density of pollinators are what would be varying. [Yan et al. (2015)](#_ENREF_25) showed that the pollen-mediated gene flow between transgenic crops and their wild relatives depends on the pollinating species (*Bombus ignitus* (10.83 %), *Apis mellifera* (5.52 %) and *Pieris rapae* (2.71 %), in addition to the distance between receiving plants and pollen donors. Unfortunately, the data does not meet the characteristics to conduct such analysis.

Over the years, many changes in the processes of management and genetic improvement of crops have occurred, especially those considered cash crops such as cotton. After 1960 a significant change was observed in the IEO, highlighting a low outcrossing rate for the following years (Figure 2). We assume that, in part, it is due to a decrease in the number of pollinators, however the use of insecticides and the region in which the studies were conducted are not significant nor comparable. In order to learn more about the causes of such difference more experiments should be carried out.

The dispersal of the species is via seeds, not pollen. Seed dispersal has been thoroughly investigated and it has shaped the evolution of the *Gossypium* genera as the species are widely spread throughout diverse geographic regions of the world. The capability of seeds to migrate ([Stephens, 1958](#_ENREF_18)) is the reason why distance is not a good measure to prevent gene flow between the species of *Gossypium* ([Wendel et al., 2009](#_ENREF_23); [Wegier et al., 2011](#_ENREF_22); [Ellstrand, 2014](#_ENREF_5)).

**Conclusion**

The data from the examined studies was adjusted to a negative exponential trend line. The IEO depends on the biological and physical reality of the studied area, as well as the shape and size of plots; however, it may be used as a first point of comparison, as is the case of our study.

**References**

Al Jibouri, H.A. (1960). Natural crossing in cotton in Iraq. *Agron Jour* 52**,** 53.

Ali, A.M., Mekki, W.H., Ibrahim, A.A., El-Siddig, K., and Babiker, E.A. (2003). "Natural outcrossing and distance of isolation in cotton (*G. hirsutum*) under Gezira conditions", in: *Proceedings to the 39th and 40th meetings of the national crop husbandry committee*, (El-Siddig, Wad Medani, Sudan).

Berkey, D.A., Savoy, B.R., Miller, S.R., and Johnson, P.G. (2002). "Pollen dissemination from adjacent fields of genetically enhanced cotton in the Mississippi delta", in: *Proceedings of the 2002 Beltwide Cotton Conferences*, (Memphis, Tennessee: National Cotton Council).

Elfawal, M.A., Bishr, M.A., and Hassoub, E.K. (1976). Natural cross pollination in Egyptian cotton (*Gossypium barbadense* L.). *J. Agric. Sci.* 86**,** 205-209. doi: 10.1017/S0021859600065151.

Ellstrand, N.C. (2014). Is gene flow the most important evolutionary force in plants? *Am. J. Bot.* 101**,** 737-753. doi: 10.3732/ajb.1400024.

Galal, H.E., Abou-El-Fittouh, H.A., and Morshed, G. (1972). Effect of direction and distance on cross pollination in Egyptian cotton (*Gossypium barbadense* L.). *Exp. Agric.* 8**,** 67-71. doi: 10.1017/S0014479700023516.

Garibaldi, L.A., Aizen, M.A., Klein, A.M., Cunningham, S.A., and Harder, L.D. (2011). Global growth and stability of agricultural yield decrease with pollinator dependence. *Proceedings of the National Academy of Sciences* 108**,** 5909-5914.

Garibaldi, L.A., Steffan-Dewenter, I., Winfree, R., Aizen, M.A., Bommarco, R., Cunningham, S.A., et al. (2013). Wild pollinators enhance fruit set of crops regardless of honey bee abundance. *science* 339**,** 1608-1611.

Kennedy, C.M., Lonsdorf, E., Neel, M.C., Williams, N.M., Ricketts, T.H., Winfree, R., et al. (2013). A global quantitative synthesis of local and landscape effects on wild bee pollinators in agroecosystems. *Ecology letters* 16**,** 584-599.

Khan, A.H., and Afzal, M. (1950). Natural crossing in cotton in western Punjab. IV. Agents of natural crossing. *Agron. J.* 42**,** 236-238.

Llewellyn, D., and Fitt, G. (1996). Pollen dispersal from two field trials of transgenic cotton in the Namoi Valley, Australia. *Mol. Breed.* 2**,** 157-166. doi: 10.1007/bf00441430.

Llewellyn, D., Tyson, C., Constable, G., Duggan, B., Beale, S., and Steel, P. (2007). Containment of regulated genetically modified cotton in the field. *Agric. Ecosyst. Environ.* 121**,** 419-429. doi: <http://dx.doi.org/10.1016/j.agee.2006.11.019>.

Loureiro, I., García-Ruiz, E., Gutiérrez, E., Gómez, P., Escorial, M.-C., and Chueca, M.-C. (2016). Pollen-mediated gene flow in the cultivation of transgenic cotton under experimental field conditions in Spain. *Ind. Crops Prod.* 85**,** 22-28. doi: <http://dx.doi.org/10.1016/j.indcrop.2016.02.045>.

Pope, O.A., Simpson, D.M., and Duncam, E.N. (1944). Effect of corn barriers on natural crossing in cotton. *J. Agric. Res.* 68**,** 347-361.

Sen, I., Oglakci, M., Bolek, Y., Cicek, B., Kisakurek, N., and Aydin, S. (2004). Assessing the out-crossing ratio, isolation distance and pollinator insects in cotton (*Gossypium hirsutum* L.). *Asian J. Plant Sci.* 3.

Simpson, D.M. (1954). Natural cross-pollination in cotton. *Technical Bulletin 1094* United States Department of Agriculture.

Simpson, D.M., and Duncan, E.N. (1956). Cotton pollen dispersal by insects. *Agron. J.* 48**,** 305-308. doi: 10.2134/agronj1956.00021962004800070007x.

Stephens, S.G. (1958). Salt water tolerance of seeds of *Gossypium* species as a possible factor in seed dispersal. *The American Naturalist* 92**,** 83-92.

Stephens, S.G., and Finkner, M.D. (1953). Natural crossing in cotton. *Econ. Bot.* 7**,** 257-269. doi: 10.1007/bf02984952.

Umbeck, P.F., Barton, K.A., Nordheim, E.V., McCarty, J.C., Parrott, W.L., and Jenkins, J.N. (1991). Degree of pollen dispersal by insects from a field test of genetically engineered cotton. *J. Econ. Entomol.* 84**,** 1943-1950. doi: 10.1093/jee/84.6.1943.

Van Deynze, A.E., Sundstrom, F.J., and Bradford, K.J. (2005). Pollen-mediated gene flow in California cotton depends on pollinator activity. *Crop Sci.* 45**,** 1565-1570. doi: 10.2135/cropsci2004.0463.

Wegier, A., Piñeyro-Nelson, A., Alarcón, J., Gálvez-Mariscal, A., Alvarez-Buylla, E.R., and Piñero, D. (2011). Recent long-distance transgene flow into wild populations conforms to historical patterns of gene flow in cotton (*Gossypium hirsutum*) at its centre of origin. *Mol. Ecol.* 20**,** 4182–4194. doi: 10.1111/j.1365-294X.2011.05258.x.

Wendel, J.F., Brubaker, C., Alvarez, I., Cronn, R., and Stewart, J.M. (2009). "Evolution and natural history of the cotton genus," in *Genetics and Genomics of Cotton,* ed. A.H. Paterson. (New York, NY: Springer US), 3-22.

Xanthopoulos, F.P., and Kechagia, U.E. (2000). Natural crossing in cotton (*Gossypium hirsutum* L.). *Aust. J. Agric. Res.* 51**,** 979-983.

Yan, S., Zhu, J., Zhu, W., Li, Z., Shelton, A.M., Luo, J., et al. (2015). Pollen-mediated gene flow from transgenic cotton under greenhouse conditions is dependent on different pollinators. *Sci. Rep.* 5**,** 15917. doi: 10.1038/srep15917.

Zhang, B.-H., Pan, X.-P., Guo, T.-L., Wang, Q.-L., and Anderson, T.A. (2005). Measuring gene flow in the cultivation of transgenic cotton (*Gossypium hirsutum* L.). *Mol. Biotechnol.* 31**,** 11-20. doi: 10.1385/mb:31:1:011.
