# Supplementary material for: The Mating System of the Wild-to-Domesticated Complex of Gossypium hirsutum L. Is Mixed
Source: Front Plant Sci. 2018 May 9;9:574. doi: 10.3389/fpls.2018.00574 (PMC5954804; doi:10.3389/fpls.2018.00574)
Supplement: Supplementary file 2 [file Data_Sheet_2.docx]

Supplementary Material 2

The mating system of the wild-to-domesticated complex of *Gossypium hirsutum* L. is mixed

**Rebeca Velázquez-López^*✚^, Ana Wegier^*✚^, Valeria Alavez, Javier Pérez-López, Valeria Vázquez-Barrios, Denise Arroyo-Lambaer, Alejandro Ponce-Mendoza, William E. Kunin**

^✚^ These authors contributed equally to this study.

^*^**Correspondence:** [rebecavelazquezl@gmail.com](mailto:rebecavelazquezl@gmail.com), [awegier@ib.unam.mx](mailto:awegier@ib.unam.mx)

**Ovule number**

To test if there were significant differences in ovule number among domesticated, introgression (SPM) and wild (YPM and CPM) plants, a Generalized Linear Mixed Model ([Zuur et al., 2009](#_ENREF_1)) was used considering the plant as a random factor, due to the fact that the number of flowers was not equal in each plant. Subsequently, a Tukey *post hoc* test was performed to evaluate the significance of results.


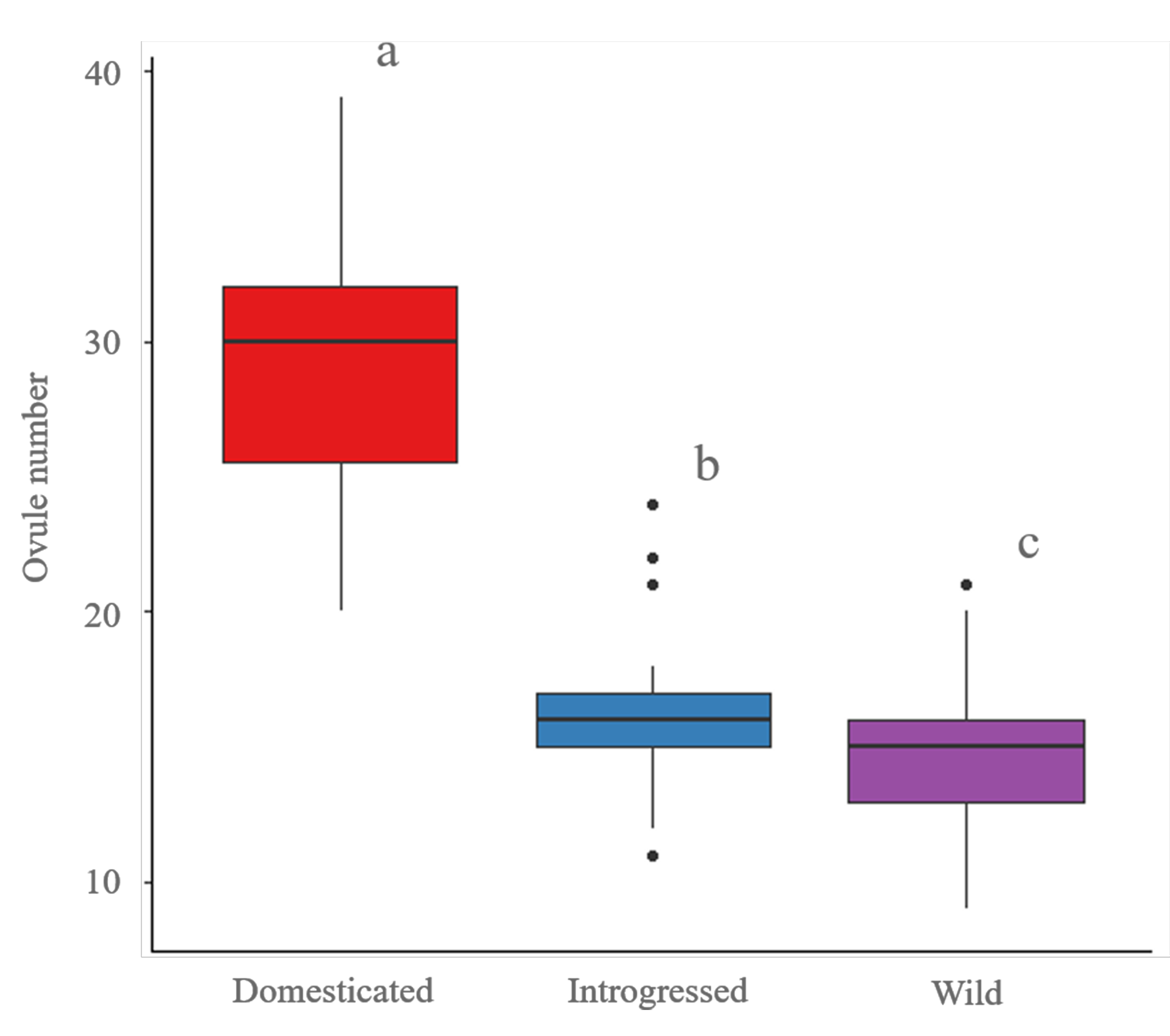
The ovule number was significantly different among treatments (P (χ^2^) = 0.001, df = 2) (Figure 1), and it was higher in domesticated than in introgressed and wild plants.

Figure 1. Box and whisker plots of ovule numbers of domesticated, introgressed (SPM) and wild (YPM and CPM) plants. The horizontal line within each box indicates the median. The bottom and top borders of the box are the first and third quartiles, respectively. The whiskers (vertical lines above and below the box) give 99 % range of the data. Different letters indicate statistically significant differences identified by the Tukey test at p < 0.05.

**References**

Zuur, A.F., Ieno, E.N., Walker, N.J., Saveliev, A.A., and Smith, G.M. (2009). "GLMM and GAMM," in *Mixed effects models and extensions in ecology with R*. (New York, NY: Springer New York), 323-341.
