# Supplementary material for: The Mating System of the Wild-to-Domesticated Complex of Gossypium hirsutum L. Is Mixed
Source: Front Plant Sci. 2018 May 9;9:574. doi: 10.3389/fpls.2018.00574 (PMC5954804; doi:10.3389/fpls.2018.00574)
Supplement: Supplementary file 3 [file Data_Sheet_3.docx]

Supplementary Material 3

The mating system of the wild-to-domesticated complex of *Gossypium hirsutum* L. is mixed

Rebeca Velázquez-López ^+^*, Ana Wegier^+^*, Valeria Alavez, Javier Pérez-López, Valeria Vázquez-Barrios, Denise Arroyo-Lambaer, Alejandro Ponce-Mendoza, William E Kunin.

^+^ These authors contributed equally to this work.

*** Correspondence:** [rebecavelazquezl@gmail.com](mailto:rebecavelazquezl@gmail.com), [awegier@ib.unam.mx](mailto:awegier@ib.unam.mx)

**Reproductive system reported in 22 crops of global importance**

In the book “Gene flow between crops and their relatives” ([Andersson and de Vicente, 2010](#_ENREF_3)), 20 crops of global importance are studied. A description of the biology of the species (center of origin, reproductive system an wild relatives) is carried out. The reproductive system of the wild and the domesticated plants is described. Given the latter, we sourced the references for each species, and with the description of the reproductive system and other references we added we have a total of 33 articles with the reproductive biology of the 22 species. For each article we focused on the reported reproductive system, the complex it is analyzing, the number of sites used, study duration, and the selfed and cross pollination descriptions. Later we constructed the table to be able to assess how the studies show how the research was performed on some of the most cultivated species in the world.

Table 1. Thirty three studies that report the reproductive biology of 22 crops of global importance.

| **Id** | **Species** | **Reproductive system** | **Complex study** | **Space (sites)** | **Time (years)** | **Seeds by Self-pollination** | **Seeds**  **by Outcrossing** | **Global conclusion of the reproductive system*** | **Reference** |
| --- | --- | --- | --- | --- | --- | --- | --- | --- | --- |
| 1 | Barley (*Hordeum vulgare*L.) | Allogamous | Domesticated | 10 | 2 | ø | ✔ | Xenogamy | [Parzies et al. (2000)](#_ENREF_26) |
| 2 | Barley (*Hordeum vulgare*L.) | Cleistogamous | Wild and domesticated | 13 | 1 | ø | ✔ | Mixed | [Abdel-Ghani et al. (2004)](#_ENREF_1) |
| 3 | Canola (*Brassica napus*L.) | Presominantly autogamous and partially allogamous | Wild and domesticated | 2 | 3 | ✔ | ✔ | Mixed | [Rakow and Woods (1987)](#_ENREF_28) |
| 4 | Canola (*Brassica napus*L.) | Presominantly autogamous and partially allogamous | Domesticated | 5 | 1 | ✔ | ✔ | Mixed | [Becker et al. (1992)](#_ENREF_4) |
| 5 | Cassava, Manioc, Yuca (*Manihot esculenta Crantz*) | Predominantly allogamous | Domesticated | 1 | 3 | ✔ | ✔ | Mixed | [Meireles da Silva et al. (2003)](#_ENREF_21) |
| 6 | Chickpea (*Cicer arietinum*L.) | Autogamous | Domesticated | 2 | 1 | ø | ✔ | Xenogamy | [Rheenen et al. (1990)](#_ENREF_30) |
| 7 | Chickpea (*Cicer arietinum*L.) | Autogamous | Domesticated and GM crop | 1 | 1 | ø | ✔ | Xenogamy | [Toker et al. (2006)](#_ENREF_33) |
| 8 | Common Bean (Phaseolus vulgaris L.) | Mixed mating system | Domesticated | 2 | 2 | ✔ | ✔ | Mixed | [Ibarra-Pérez et al. (1997)](#_ENREF_13) |
| 9 | Common Bean (Phaseolus vulgaris L.) | Allogamous | Domesticated | 2 | 5 | ø | ✔ | Xenogamy | [Chaves-Barrantes et al. (2014)](#_ENREF_8) |
| 10 | Cotton (*Gossypium hirsutum*) | Autogamous | Domesticated | 11 | 1 | ø | ✔ | Xenogamy | [Meredith and Bridge (1973)](#_ENREF_22) |
| 11 | Cotton (*Gossypium hirsutum*) | Allogamous | Domesticated | 12 | 1 | ø | ✔ | Xenogamy | [Simpson (1954)](#_ENREF_31) |
| 11 | Cotton (*Gossypium hirsutum*) | Allogamous | Domesticated | 15 | 1 | ø | ✔ | Xenogamy | [Simpson (1954)](#_ENREF_31) |
| 11 | Cotton (*Gossypium hirsutum*) | Allogamous | Domesticated | 15 | 1 | ø | ✔ | Xenogamy | [Simpson (1954)](#_ENREF_31) |
| 11 | Cotton (*Gossypium hirsutum*) | Allogamous | Domesticated | 6 | 1 | ø | ✔ | Xenogamy | [Simpson (1954)](#_ENREF_31) |
| 12 | Cowpea (*Vigna unguiculata* (L.) Walp.) | Predominantly autogamous | Wild and  domesticated | 2 | 1 | ø | ✔ | Xenogamy | [Rachie et al. (1975)](#_ENREF_27) |
| 13 | Cowpea (*Vigna unguiculata* (L.) Walp.) | Allogamus | Wild | 2 | 1 | ✔ | ✔ | Mixed | [Lush (1979)](#_ENREF_20) |
| 14 | Finger Millet (Eleusine coracana (L.) Gaertn.) | Self-fertilizing and cross-pollination | Wild and domesticated | 1 | 1 | ✔ | ✔ | Mixed | [Dodake and Dhonukshe (1998)](#_ENREF_9) |
| 15 | Maize, Corn (Zea mays L.) | Allogamous, typically cross-pollination | Domesticated | 1 | 10 | ✔ | ✔ | Mixed | [Sleper and Poehlman (2006)](#_ENREF_32) |
| 16 | Oat (*Avena sativa*L.) | Predominantly Self-pollinating | Wild | 7 | 3 | ✔ | ✔ | Mixed | [Imam and Allard (1965)](#_ENREF_14) |
| 17 | Oat (*Avena sativa*L.) | Predominantly Self-pollinating | Wild | 1 | 1 | ø | ✔ | Xenogamy | [Murray et al. (2002)](#_ENREF_23) |
| 18 | Peanut, Groundnut (*Arachis hypogaea*L.) | Predominantly autogamous | Wild and domesticated | 1 | 3 | ✔ | ✔ | Mixed | [Knauft et al. (1992)](#_ENREF_15) |
| 19 | Pearl Millet (*Pennisetum glaucum* (L.) R. Br.) | Mainly allogamous | Domesticated | 1 | 1 | ø | ✔ | Xenogamy | [Leuck and Burton (1966)](#_ENREF_19) |
| 20 | Pigeonpea (*Cajanus cajan*(L.) Millsp.) | Mainly self-fertilizing, cross-pollination and frequently out-crossing | Domesticated | 13 | 1 | ✔ | ✔ | Mixed | [Bhatia et al. (1980)](#_ENREF_5) |
| 21 | Pigeonpea (*Cajanus cajan*(L.) Millsp.) | Self-fertilizing and cross-pollination | Domesticated | 6 | 1 | ✔ | ✔ | Mixed | [Onim (1980)](#_ENREF_25) |
| 22 | Potato (*Solanum tuberosum*L.) | Mixed mating system | Domesticated | 1 | 1 | ✔ | ✔ | Mixed | [Brown (1993)](#_ENREF_6) |
| 23 | Rice (*Oryza sativa* L.) | Cleistogamous | Domesticated | 1 | 1 | ✔ | ✔ | Mixed | [Kolhe and Bhat (1981)](#_ENREF_16) |
| 24 | Sorghum (Sorghum bicolor (L) Moench) | Predominantly autogamous | Domesticated | 2 | 3 | ø | ✔ | Xenogamy | [Ellstrand and Foster (1983)](#_ENREF_10) |
| 24 | Soybean (*Glycine max*(L.)Merr.) | Autogamous and natural out-crossing | Domesticated | 1 | 2 | ✔ | ✔ | Mixed | [Ahrent and Caviness (1994)](#_ENREF_2) |
| 25 | Soybean (*Glycine max*(L.) Merr.) | Self-pollination and natural out-crossing | Domesticated | 1 | 3 | ø | ✔ | Mixed | [Caviness (1966)](#_ENREF_7) |
| 26 | Soybean (*Glycine max*(L.) Merr.) | Highly autogamous and natural out-crossing | Domesticated | 1 | 2 | ø | ✔ | Xenogamy | [Ray et al. (2003)](#_ENREF_29) |
| 28 | Sweetpotato (*Ipomoea batatas*(L.) Lam.) | Allogamous | Domesticated | 1 | 1 | ø | ✔ | Xenogamy | [Epperson and Clegg (1987)](#_ENREF_11) |
| 29 | Wheat,Bread Wheat (*Triticum aestivum*L.) | Mainly Self-pollinating | Wild | 1 | 2 | ø | ✔ | Xenogamy | [Tsegaye (1996)](#_ENREF_34) |
| 30 | Wheat,Bread Wheat (*Triticum aestivum*L.) | Mainly autogamous | Domesticated | 1 | 2 | ø | ✔ | Xenogamy | [Hucl (1996)](#_ENREF_12) |
| 31 | Banana and Plantain (*Musa*spp.) | Autogamous and Allogamous | Wild and domesticated | 3 | 1 | ✔ | ✔ | Mixed | [Nur (1976)](#_ENREF_24) |
| 32 | Carrots (*Daucus carota* L.) | Xenogamy and Geitonogamy | Wild and domesticated | 1 | 1 | ø | ø | Geitonogamy and xenogamy | [Koul et al. (1989)](#_ENREF_17) |
| 33 | Fennel (*Foeniculum vulgare* Mill.) | Xenogamy and Geitonogamy | Wild and domesticated | 2 | 1 | ✔ | ✔ | Mixed | [Koul et al. (1996)](#_ENREF_18) |

*This column contains the biological capacity of the species, ignoring the local conditions in which it can be measured depending on the research questions.

**References**

Abdel-Ghani, A.H., Parzies, H.K., Omary, A., and Geiger, H.H. (2004). Estimating the outcrossing rate of barley landraces and wild barley populations collected from ecologically different regions of Jordan. *Theor. Appl. Genet.* 109**,** 588-595. doi: 10.1007/s00122-004-1657-1.

Ahrent, D.K., and Caviness, C.E. (1994). Natural cross-pollination of twelve soybean cultivars in Arkansas. *Crop Sci.* 34**,** 376-378.

Andersson, M.S., and de Vicente, M.C. (2010). *Gene flow between crops and their wild relatives.* Baltimore: John Hopkins University Press.

Becker, H., Damgaard, C., and Karlsson, B. (1992). Environmental variation for outcrossing rate in rapeseed (*Brassica napus*). *Theor. Appl. Genet.* 84**,** 303-306.

Bhatia, G.K., Gupta, S.C., Green, J.M., and Sharma, D. (1980). "Estimates of natural cross-pollination in *Cajanus cajan* (L.) Millsp.: several experimental approaches", in: *Proceedings of the International Workshop on Pigeonpeas*, ed. Y.L. Nene (Patancheru, India: ICRISAT Center), 129-136.

Brown, C.R. (1993). Outcrossing rate in cultivated autotetraploid potato. *Am. J. Potato Res.* 70**,** 725-734.

Caviness, C.E. (1966). Estimates of natural crosspollination in Jackson soybeans in Arkansas. *Crop Sci.* 6**,** 211-212. doi: 10.2135/cropsci1966.0011183X000600020034x.

Chaves-Barrantes, N.F., Araya-Villalobos, R., and Debouck, D.G. (2014). Cruzamiento natural en frijol común en Costa Rica. *Agron. Mesoam.* 25**,** 23-33.

Dodake, S., and Dhonukshe, B. (1998). Variability in floral structure and floral biology of finger millet (*Eleusine coracana* (L.) Gaertn.). *Indian J. Genet. Plant Breed.* 58**,** 107-112.

Ellstrand, N.C., and Foster, K.W. (1983). Impact of population structure on the apparent outcrossing rate of grain sorghum (*Sorghum bicolor*). *Theor. Appl. Genet.* 66**,** 323-327. doi: 10.1007/bf00251167.

Epperson, B.K., and Clegg, M.T. (1987). First-pollination primacy and pollen selection in the morning glory, *Ipomoea purpurea*. *Heredity* 58**,** 5-14. doi: 10.1038/hdy.1987.2.

Hucl, P. (1996). Out-crossing rates for 10 Canadian spring wheat cultivars. *Can. J. Plant Sci.* 76**,** 423-427. doi: 10.4141/cjps96-075.

Ibarra-Pérez, F.J., Ehdaie, B., and Waines, J.G. (1997). Estimation of outcrossing rate in common bean. *Crop Sci.* 37**,** 60-65. doi: 10.2135/cropsci1997.0011183X003700010009x.

Imam, A.G., and Allard, R.W. (1965). Population studies in predominantly self-pollinated species. VI. Genetic variability between and within natural populations of wild oats from differing habitats in California. *Genetics* 51**,** 49-62.

Knauft, D.A., Chiyembekeza, A.J., and Gorbet, D.W. (1992). Possible reproductive factors contributing to outcrossing in peanut (*Arachis hypogaea* L.). *Peanut Sci.* 19**,** 29-31. doi: 10.3146/i0095-3679-19-1-7.

Kolhe, G.L., and Bhat, N.R. (1981). Genetic study of cleistogamy in rice (*Oryza sativa* L.). *Curr. Sci.* 50**,** 419-420.

Koul, P., Koul, A.K., and Hamal, I.A. (1989). Reproductive biology of wild and cultivated carrot (*Daucus carota* L.). *New Phytol.* 112**,** 437-443.

Koul, P., Sharma, N., and Koul, A.K. (1996). Reproductive biology of wild and cultivated fennel (*Foeniculum vulagre* Mill.). *Proc. Indian natn. Sci. Acad.* B62**,** 125-134.

Leuck, D.B., and Burton, G.W. (1966). Pollination of pearl millet by insects. *J. Econ. Entomol.* 59**,** 1308-1309. doi: 10.1093/jee/59.5.1308.

Lush, W.M. (1979). Floral morphology of wild and cultivated cowpeas. *Econ. Bot.* 33**,** 442-447. doi: 10.1007/bf02858340.

Meireles da Silva, R., Bandel, G., and Sodero Martins, P. (2003). Mating system in an experimental garden composed of cassava (*Manihot esculenta* Crantz) ethnovarieties. *Euphytica* 134**,** 127-135. doi: 10.1023/B:EUPH.0000003644.60126.4a.

Meredith, W.R., and Bridge, R.R. (1973). Natural crossing in cotton (*Gossypium hirsutum* L.) in the Delta of Mississippi. *Crop Sci.* 13**,** 551-552. doi: 10.2135/cropsci1973.0011183X001300050016x.

Murray, B.G., Morrison, I.N., Friesen, L.F., Murray, B.G., Morrison, I.N., and Friesen, L.F. (2002). Pollen-mediated gene flow in wild oat. *Weed Sci.* 50**,** 321-325. doi: 10.1614/0043-1745(2002)050[0321:PMGFIW]2.0.CO;2.

Nur, N. (1976). Studies on pollination in Musaceae. *Ann. Bot.* 40**,** 167-177.

Onim, J.F.M. (1980). "Pigeonpea improvment research in Kenya", in: *Proceedings of the International Workshop on Pigeonpeas*, ed. Y.L. Nene (Patancheru, India: ICRISAT Center), 427-449.

Parzies, H.K., Spoor, W., and Ennos, R.A. (2000). Outcrossing rates of barley landraces from Syria. *Plant Breed.* 119**,** 520-522. doi: 10.1046/j.1439-0523.2000.00532.x.

Rachie, K.O., Rawal, K., Franckowiak, J.D., and Akinpelu, M.A. (1975). Two outcrossing mechanisms in cowpeas, *Vigna unguiculata* (L.) Walp. *Euphytica* 24**,** 159-163. doi: 10.1007/bf00147180.

Rakow, G., and Woods, D.L. (1987). Outcrossing in rape and mustard under Saskatchewan paririe conditions. *Can. J. Plant Sci.* 67**,** 147-151. doi: 10.4141/cjps87-017.

Ray, J.D., Kilen, T.C., Abel, C.A., and Paris, R.L. (2003). Soybean natural cross-pollination rates under field conditions. *Environ. Biosafety Res.* 2**,** 133-138.

Rheenen, H.A.v., Gowda, C.L.L., and Janssen, M.G. (1990). Natural cross-fertilization in chickpea (*Cicer arietinum* L.). *Indian J. Genet. Plant Breed.* 50**,** 329-332.

Simpson, D.M. (1954). Natural cross-pollination in cotton. *Technical Bulletin 1094* United States Department of Agriculture.

Sleper, D.A., and Poehlman, J.M. (2006). *Breeding field crops.* Oxford: Blackwell publishing.

Toker, C., Canci, H., and Ceylan, F.O. (2006). Estimation of outcrossing rate in chickpea (*Cicer arietinum* L.) sown in autumn. *Euphytica* 151**,** 201-205. doi: 10.1007/s10681-006-9140-5.

Tsegaye, S. (1996). Estimation of outcrossing rate in landraces of tetraploid wheat (*Triticum turgidum* L.). *Plant Breed.* 115**,** 195-197. doi: 10.1111/j.1439-0523.1996.tb00901.x.
